# Hydrogen Peroxide-Induced DNA Damage and Repair through the Differentiation of Human Adipose-Derived Mesenchymal Stem Cells

**DOI:** 10.1155/2018/1615497

**Published:** 2018-10-10

**Authors:** Mahara Valverde, Jonathan Lozano-Salgado, Paola Fortini, Maria Alexandra Rodriguez-Sastre, Emilio Rojas, Eugenia Dogliotti

**Affiliations:** ^1^Instituto de Investigaciones Biomédicas, Universidad Nacional Autónoma de México, C.U. 04510, Mexico; ^2^Department of Environment and Health, Istituto Superiore di Sanità, Viale Regina Elena 299, 00161 Roma, Italy

## Abstract

Human adipose-derived mesenchymal stem cells (hADMSCs) are recognized as a potential tool in cell tissue therapy because of their capacity to proliferate and differentiate in vitro. Several studies have addressed their use in regenerative medicine; however, little is known regarding their response to DNA damage and in particular to the reactive oxygen species (ROS) that are present in the microenvironment of implantation. In this study, we used the ROS-inducing agent hydrogen peroxide to explore the responses of (1) hADMSCs and (2) derived terminally differentiated adipocytes to oxidatively generated DNA damage. Using single cell gel electrophoresis, a dose-related increase was found for both DNA breaks and oxidative lesions (formamidopyrimidine DNA glycosylase-sensitive sites) upon exposure of hADMSCs to hydrogen peroxide. DNA repair capacity of hADMSCs was affected in cells exposed to 150 and 200 *μ*M of hydrogen peroxide. An increase in the basal levels of DNA breaks and oxidative DNA lesions was observed through adipocyte differentiation. In addition, hydrogen peroxide-induced DNA damage increased through adipocyte differentiation; DNA repair capacity also decreased. This study is the first follow-up report on DNA repair capacity during adipogenic differentiation. Remarkably, in terminally differentiated adipocytes, DNA breakage repair is abolished while the repair of DNA oxidative lesions remains efficient.

## 1. Introduction

Before the 1980s, adipose tissue was considered only a passive energy storage reservoir. However, when its participation in the metabolism of sex hormones was confirmed, adipose tissue was recognized as an important endocrine organ [1]. A substantial breakthrough occurred when a new source of adult stem cells, called adipose-derived stem cells, was described for the first time by Zuk et al. [2] as self-renewing multipotent cells [3]. Human adipose-derived mesenchymal stem cells (hADMSCs) are isolated from adipose (fat) tissues by lipoaspiration or biopsy. Lipoaspirates represent a heterogeneous mixture of cell types, including adipocytes, endothelial cells, smooth muscle, pericytes, and progenitor cells [2]. Such properties make hADMSCs a potential tool for therapy in clinics [4] and, in particular, for autologous cell transplantation. However, the low survival rate and increased cell death after implantation into injured tissue suggest that hADMSCs are damaged by the local microenvironment likely due to sustained oxidative stress with the overproduction of reactive oxygen species (ROS) [5, 6]. ROS are well known to play a role in the growth and homeostasis of MSCs even when they are generated as byproducts of normal energy metabolism. Chemically, hydrogen peroxide (H_2_O_2_) is a poorly reactive ROS in the absence of ions of transition metals; however, because in biological systems it is ubiquitously produced and presents a relatively long half-life, H_2_O_2_ fulfills the prerequisites for serving as an intracellular messenger and acting as a cell signaling molecule [7]. In chemical terms, H_2_O_2_ can act as a mild oxidizing agent or as a mild reducing agent, but it does not readily oxidize most biological molecules, including lipids, DNA, and proteins, unless the latter have hyperreactive thiol groups or methionine residues [8]. Because of its mode of action, H_2_O_2_ can induce distinct responses depending on the cell type. In this study, we explored the capacity of H_2_O_2_ to induce DNA damage and repair in hADMSCs when proliferating and in different stages of adipocyte differentiation. Although all living cells are provided with a plethora of DNA repair mechanisms to address different DNA lesions and to preserve genomic integrity, mesenchymal stem cells are expected to have a strong response to DNA damage because of their remarkable abilities of self-renewal and differentiation into different functional cell types. In the present study, we specifically investigated whether the response to oxidatively generated DNA damage is modulated during the adipogenic differentiation of hADMSCs. We show that hADMSCs repair DNA breaks and alkali labile sites very efficiently when proliferating but that their repair capacity declines during adipocyte differentiation. Interestingly, adipocytes maintain their ability to repair formamidopyrimidine DNA glycosylase (Fpg) sites, including 7,8-dihydro-8-oxoguanine (8-oxoguanine), 8-oxoadenine, aflatoxin B1-fapy-guanine, 5-hydroxy-cytosine, and 5-hydroxy-uracil [9, 10].

## 2. Material and Methods

### 2.1. hADMSC Cell Culture

Normal hADMSCs (ATCC® PCS-500-011™) were cultured at an early passage (passage 4). Cell cultures were made using Gibco MesenPRO RS™ Medium (cat. number 12746-012) prepared according to the supplier's specifications and incubated at 37°C and 5% CO_2_. Medium changes were performed every 48 hours and treated at ~85% confluence. For harvesting, the cultures were treated with 0.05% Trypsin-EDTA (Gibco, cat. number 25300-054) at 37°C and 5% CO_2_ for 3 minutes (sufficient time to obtain a total suspension of the culture). They were then transferred to PBS and centrifuged at 300 ×g for 5 minutes. They were subsequently handled depending on the test to be performed.

### 2.2. hADMSC Surface Markers

Anti-CD45, anti-CD146, anti-CD105, and anti-CD90 (Human Mesenchymal Stem Cell Multi-Color Flow Cytometry Kit, R&D Systems; cat. number FMC002) were used for immunophenotype determination of hADMSCs before and after adipocyte differentiation, following the recommendations of the International Society for Cellular Therapy [11]. The protocol indicated by the provider was followed; cell samples were washed with staining buffer and then blocked. The antibody or the corresponding isotype control antibody was then added. Incubation was carried out by 30 minutes at room temperature in the dark, and any excess antibody was removed by washing with staining buffer before analysis. The acquisitions were performed on a Blue/Red Attune (BD Biosystems) flow cytometer.

### 2.3. Hydrogen Peroxide Treatment

hADMSCs were treated at a density of 4000/cm^2^. H_2_O_2_ bolus treatment (0, 50, 100, 150, and 200 *μ*M) lasted for 2 hours, and the recovery time in fresh medium was 24 h posttreatment. To be precise, the corresponding incipient toxic doses of H_2_O_2_ were 0, 152.5, 305, 457.5, and 600 nmol of H_2_O_2_/mg cell protein.

Cells at different differentiation durations (days 6, 12, and 14) were treated for 2 hours with H_2_O_2_ 100 *μ*M (305 nmol/mg cell protein), and the recovery time was 24 h posttreatment in fresh medium.

Cells were harvested with trypsin-EDTA; the cell suspension obtained was used to perform the lysosomal activity test, determination of DNA damage (breaks and oxidative lesions), and DNA repair capacity.

### 2.4. Lysosomal Activity Test

A lysosomal activity test was used to determine cell viability and was performed using the FDA-Et-Br (fluorescein diacetate-ethidium bromide) stain. The cell suspension was mixed 1 : 1 with stain solution, and the analysis was performed by fluorescence microscopy (Olympus BMX-60 with a UM61002 filter). FDA is taken up by cells that through esterase activity transform nonfluorescent FDA into a green fluorescent metabolite. The nuclei of the dead cells are then stained with ethidium bromide and visualized as red fluorescence.

### 2.5. Reactive Oxygen Species

This technique is based on the ROS-dependent oxidation of dihydrorhodamine 123 (DHR123, Calbiochem, cat. number 309825) to rhodamine 123 (Sigma-Aldrich, cat. number R-8004). hADMSCs or adipocyte cells were grown under the required conditions. The medium was then removed and washed with PBS. Cells were harvested and counted in a Moxi automated cell counter (ORFLO, Montana, US). Aliquots equivalent to 2 × 10^5^ cells were collected and centrifuged at 1200 rpm for 5 minutes. The supernatant was poured, and 180 *μ*l of buffer A (140 mM NaCl, 5 mM KCl, 0.8 mM MgSO_4_·7H_2_O, 1.8 mM CaCl_2_, 5 mM glucose, and 15 mM HEPES) and 20 *μ*l of dihydrorhodamine 123 (1 mM) were added to the pellet. This mixture was placed in a 96-well plate and read using a fluorescence reader (BioTek FLx8000) at a wavelength of 505 nm. The results were interpolated in a curve of rhodamine 123 in buffer A at concentrations of 0–10 *μ*M. Data were expressed as nmol of rhodamine 123/2 × 10^5^ cells.

### 2.6. DNA Breaks and DNA Oxidative Damage

DNA damage was determined by alkaline single cell gel electrophoresis assay to evaluate the presence of DNA breaks produced, which includes single and double strand breaks, as well as alkali labile sites in hADMSC cells either proliferating or during differentiation (control, H_2_O_2_ treatment, and 24 h posttreatment) [12, 13]. For each experimental condition, at least 10,000 cells were mixed with 75 *μ*l of 0.5% low melting point (LMP) agarose. The cells were loaded onto microscope slides prelayered with 200 *μ*l of 0.5% normal melting point agarose and covered with a third layer of LMP agarose 0.5%. Briefly, after lysis of the cells at 4°C for at least 1 h in a buffer consisting of 2.5 M NaCl, 100 mM EDTA, and 10 mM Tris, pH 10, supplemented with 10% DMSO and 1% Triton X-100, the slides were placed in a horizontal electrophoresis chamber with running buffer solution (300 mM NaOH, 1 mM Na_2_EDTA, pH > 13). The slides remained in the electrophoresis buffer for 20 minutes to allow the DNA to unwind and reveal alkali-labile sites (AP sites). Electrophoresis was performed for 20 min at 300 mA and 25 V, ~0.8 V/cm. All steps were performed in the dark to avoid direct light. After electrophoresis, the slides were gently removed and rinsed with neutralization buffer (0.4 M Tris, pH 7.5) at room temperature for 15 min, dehydrated with absolute ethanol for 15 min, and air-dried. Ethidium bromide (20 *μ*l of 20 mg/ml solution) was added to each slide and a coverslip was placed on the gel. Individual cells were visualized at 20x magnification with an Olympus BX-60 microscope with fluorescence attachments (515–560 nm excitation filter, 590 nm barrier filter), and the DNA damage was determined using Komet 5.0 software (Kinetic Imaging Ltd.). To evaluate DNA migration, 50 nucleoids per slide (300 nucleoids total per condition) were scored for each experimental condition. The data were divided into five categories according to the Olive tail moment (OTM) score. The total number of nucleoids in each category was counted and multiplied by an assigned value of 0–4 according to the damage class. The sum of all the categories was calculated and considered the damage index. The overall score was expected to vary between 0 and 400 arbitrary units. Alternatively, the OTM score obtained by the software was employed.

DNA oxidative damage was identified via incubation with formamidopyrimidine DNA glycosylase (Fpg) to reveal 7,8-dihydro-8-oxoguanine (8-oxoguanine), 8-oxoadenine, aflatoxin B1-fapy-guanine, 5-hydroxy-cytosine, and 5-hydroxy-uracil [9, 10]. Briefly, after treatment, the cells immersed in LMP agarose 1% were layered on microscope slides precoated with 1% normal melting point agarose and immersed in lysis buffer for at least 1 h at 4°C. The slides were then rinsed with buffer solution (50 mM Tris-base, 10 mM EDTA, pH 7.6) for 5 minutes. Oxidative lesions were digested by Fpg (Trevigen, CA, USA), under ideal conditions of pH and temperature suggested by the provider. Coverslips were placed on the slides and were incubated for 30 min at 37°C in a humidified atmosphere. A set of slides with cells of every experimental condition in buffer (without enzyme) was included to confirm that the DNA strand breaks were enzyme specific. Following enzyme incubation, the slides were rinsed with solution buffer (50 mM Tris-base, 200 mM EDTA, pH 7.6) and subjected to conventional comet electrophoresis (~0.8 V/cm) for 20 min without unwinding incubation. Dehydration, stain, and analysis were performed as previously described.

To determine the DNA repair capacity, we apply

(1) $$\begin{array}{c} \%\;DNA\;repair\;capacity=\frac{\left[remaining\;DNA\;damage\;\left(OTM\right)\times 100\right]}{net‐induced\;DNA\;damage\;\left(OTM\right)},\\ Remaining\;damage=DNA\;damage\;induced\;by\;H_{2}O_{2}\;\left(OTM\right)–posttreatment\;DNA\;damage\;\left(OTM\right),\\ Net\;induced\;DNA\;damage=DNA\;damage\;induced\;by\;H_{2}O_{2}\;\left(OTM\right)–DNA\;damage\text{ of }control. \end{array}$$

### 2.7. Adipocyte Differentiation

Cultures of hADMSCs (pass 4) reached ~80% confluence. They were then passaged into a plate at a density of 18,000 cells/cm^2^ in MesenPRO RS™ Medium Gibco (cat. number 12746-012). The cells were incubated at 37°C and 5% CO_2_ for 48 h before initiating differentiation. Thereafter, a wash with PBS was performed to remove components from the previous medium and adding the StemPro® Adipogenesis Differentiation Kit, Gibco (cat. number A10070-01). The media changes were established every 72 h and incubated at 37°C and 5% CO_2_ until day 14 of adipocyte differentiation. For harvest, the cultures at day 6, 12, and 14 were treated with 0.25% Trypsin-EDTA (Gibco, cat. number 25200-056) at 37°C and 5% CO_2_ for 3 minutes. With the help of a plastic scraper, a culture suspension was obtained, which was transferred to PBS to be centrifuged at 300 ×g for 5 minutes. They were subsequently handled depending on the test to be performed.

### 2.8. hFATP-1

Anti-hFATP-1 (human fat acid transporter protein 1) (R&D Systems; cat. number IC3304P) was used as the surface marker of adipocyte differentiation [14]. Determination of hFATP-1 before and after adipocyte differentiation. The protocol indicated by the provider was followed. A solution of saponin (0.1% w/v saponin and 0.05% NaN_3_ in PBS) was used for permeabilization. The antibody or the corresponding isotype control antibody was then added. Incubation was carried out by 30 minutes at room temperature in the dark, and any excess antibody was removed by washing with staining buffer before analysis. The acquisitions were performed on a Blue/Red Attune (BD Biosystems) flow cytometer.

### 2.9. Cell Cycle Analysis

The cell cycle was evaluated using a DNA intercalating agent (propidium iodide; IP) and RNase A to decrease nonspecificity (Muse Cell Cycle Kit, cat. number MCH100106). After harvesting, the cells were washed with PBS and then fixed with 70% ethanol for at least 48 h. The cells were then centrifuged 300 ×g for 5 minutes and washed with PBS. The IP was added and incubated for 30 minutes at room temperature in the dark. Finally, the acquisition was performed using a FACScan cytometer (BD Biosystems) from the cytofluorometry unit of the Instituto de Investigaciones Biomédicas, UNAM. The cell cycle phase of the cell populations was then determined according to the deoxyribonucleic content.

### 2.10. Oil Red O Stain

To confirm adipocyte differentiation, the hADMSCs were seeded in 12-well plates. Differentiation was initiated when confluence was 80%. The corresponding stains were carried out at day 14 of differentiation. The cells were washed twice with PBS and then fixed with 10% paraformaldehyde for 30 minutes at room temperature. Finally, two washes were performed with PBS, and Oil Red O (Trevigen cat. number 5010-024-05) prepared according to the supplier was added followed by incubation for 30 minutes with gentle shaking and protection from light. Two additional washes were then carried out, and PBS was added for the inverted microscope display (Olympus IX50-S8F2). For quantification, the PBS was then removed, and the dye was extracted with isopropanol and incubated for 10 minutes with shaking and protection from light. The supernatant was finally placed in a quartz cell (Quartz Spectrophotometer Cell Semi Micro, 9-Q-10 mm, Bio-Rad Laboratories), and the absorbance of the sample was measured on a spectrophotometer (Ultrospec 3000, Pharmacia Biotech) at 500 nm using isopropanol as a blank to obtain the relative lipid accumulation.

### 2.11. Statistical Analysis

Statistical analysis was performed using the software SigmaStat 3.5 and SigmaPlot 10.0. Nonparametric tests were used for single cell gel electrophoresis analyses, Kruskal-Wallis (one-way ANOVA on ranks) was used for all pairwise multiple comparison procedures (Dunn's test), and for data with normal distribution, Student's *t*-tests were performed to assess lysosomal activity, ROS levels, surface markers, and the cell cycle.

## 3. Results

### 3.1. hADMSC Immunophenotype and Characterization of Adipocyte Differentiation

**Figure 1:**
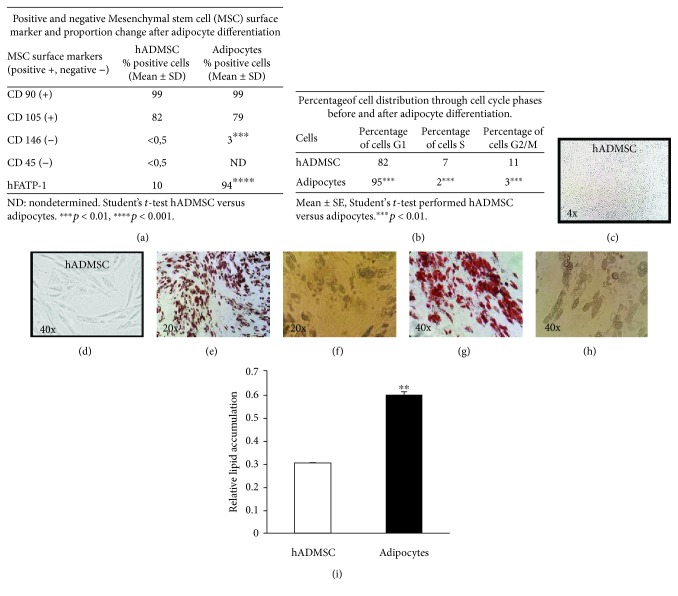
hADMSC immunophenotype and characterization of adipocyte differentiation. Positive and negative surface markers of MSCs and adipocyte differentiation marker hFATP-1 in (a). Cell cycle and G1 arrest of adipocytes in (b). hADMSC representative morphology at 4x magnification in (c) and 40x magnification in (d). Adipocyte differentiation after 14 days evidenced by Oil Red O staining. (e) and (g) are representative images at 20x and 40x magnification, respectively. Light microscopy images at day 14 of differentiation are represented in (f) and (h), corresponding to 20x and 40x magnification, respectively. The increase in the relative lipid accumulation between hADMSCs and adipocytes was determined by Student's *t*-test, ^∗^ *p* < 0.05 (i).

Mesenchymal stem cells (MSCs) are functionally defined by their capacity to self-renew and their ability to differentiate into multiple cell types (adipocytes, chondrocytes, and osteocytes), which are phenotypically characterized as positive for CD105 and CD90 and negative for CD146 and CD45. The percentage of cells that present these characteristic surface markers are shown in Figure 1(a). The percentage of cells positive for the human fatty acid transporter protein 1 (hFATP1) is also presented to show the tissue source as well as the degree of adipogenic differentiation. The distribution of cells along the cell cycle further confirm that an efficient differentiation is achieved as shown by the increase in the percentage of cells in G1 and a decrease in the percentage of cells in the S and G2/M phases in adipocytes compared with the undifferentiated cells (hADMSCs) (Figure 1(b)). In addition, the relative lipid accumulation as measured by body fat stain with Oil Red O (Figures 1(e)–1(h)) shows lipid accumulation after 14 days (when adipocyte differentiation is complete) compared with that of hADMSCs (Figure 1(i)).

### 3.2. H_2_O_2_-Induced DNA Damage and Repair Capacity in hADMSCs

**Figure 2:**
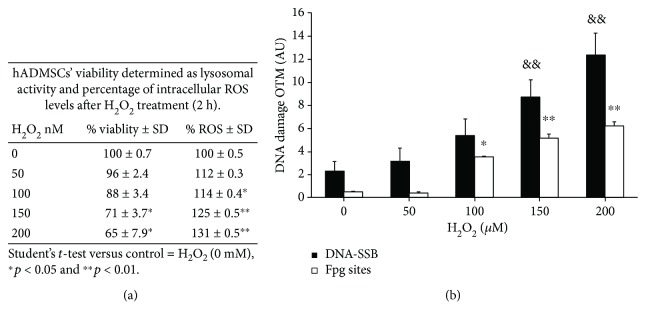
H_2_O_2_ dose response in human adipose-derived mesenchymal stem cells. Percentage of cell viability and reactive oxygen species (ROS) in human adipose-derived mesenchymal stem cells (hADMSC) (a). DNA damage induced after 2 h of hydrogen peroxide, expressed as Olive tail moment (OTM) and corresponding to DNA breaks and alkali labile sites (DNA-SSB) and Fpg sites (7,8-dihydro-8-oxoguanine (8-oxoguanine), 8-oxoadenine, aflatoxin B1-fapy-guanine, 5-hydroxy-cytosine, and 5-hydroxy-uracil) in (b) (mean ± SD) (Supplementary Figure 1, comet assay images). To be precise, the corresponding incipient toxic doses of H_2_O_2_ were 0, 152.5, 305, 457.5, and 600 nmol of H_2_O_2_/mg cell protein. Data represent 3 independent duplicate experiments (*N* = 3). Nonparametric tests were performed using the Kruskal-Wallis test (one-way ANOVA on ranks), and all pairwise multiple comparison procedures were performed with Dunn's test. DNA-SSB difference versus control (0 *μ*M or 0 nmol/mg cell protein), ^&&^ *p* < 0.01. Fpg site difference versus control (0 *μ*M), ^∗∗^ *p* < 0.01.

**Figure 3:**
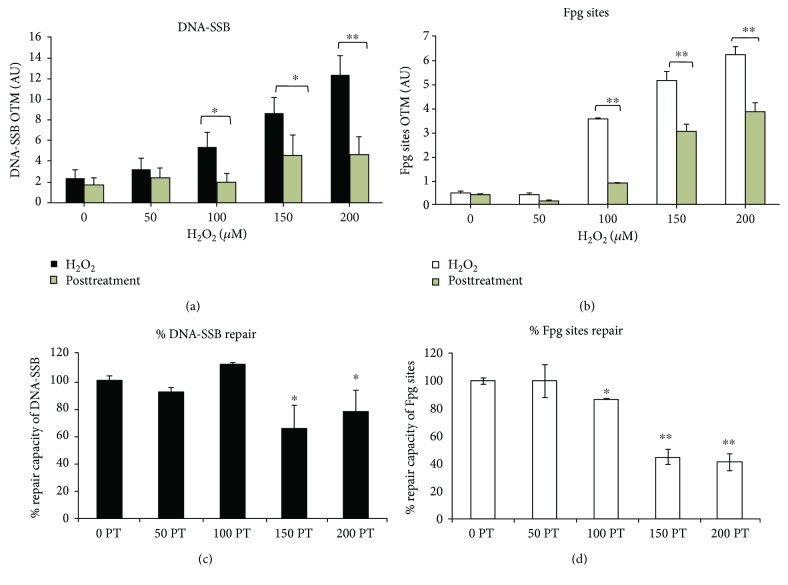
DNA repair capacity of H_2_O_2_ treatment in human adipose-derived mesenchymal stem cells (hADMSCs). DNA damage induced and remnant after 24 h posttreatment (PT), expressed as Olive tail moment (OTM) corresponding to DNA breaks and alkali labile sites in (a) (mean ± SD). DNA-Fpg sites (7,8-dihydro-8-oxoguanine (8-oxoguanine), 8-oxoadenine, aflatoxin B1-fapy-guanine, 5-hydroxy-cytosine, and 5-hydroxy-uracil) in (b) (mean ± SD). Percentage of DNA repair capacity of DNA breaks and alkali labile sites in (c) (percentage ± SD) and percentage of DNA-Fpg sites' repair capacity in (d) (percentage ± SD). 0 PT = control PT; 50 PT = 50 *μ*M H_2_O_2_ PT; 100 PT = 100 *μ*M H_2_O_2_ PT; 150 PT = 150 *μ*M H_2_O_2_ PT; and 200 PT = 200 *μ*M H_2_O_2_ PT. All data were obtained in 3 independent duplicate experiments (*N* = 3). Kruskal-Wallis was performed with one-way ANOVA on ranks, and all pairwise multiple comparison procedures were performed with Dunn's test. Differences between induced damage and remnant damage were obtained per (a) and (b), ^∗^ *p* < 0.05 and ^∗∗^ *p* < 0.01. Student's *t*-test versus control was applied for data in (c) and (d), ^∗^ *p* < 0.05 and ^∗∗^ *p* < 0.01. Images in Supplementary Figure 3.

hADMSCs exposed to H_2_O_2_ for 2 h showed a dose-dependent response in the metabolic viability test as well as the ROS level assay. In particular, a slight decrease in esterase activity was observed at concentrations higher than 100 *μ*M and was paralleled by an increase in the ROS level (Figure 2(a)). The DNA damage induced by H_2_O_2_ as both DNA breaks and alkali labile sites as well as oxidative lesions shows a clear dose-dependent response (Figure 2(b)). A relatively higher induction of DNA breaks and alkali labile sites is observed with respect to oxidative DNA lesions. DNA damage may lead to genome instability when it is not repaired. We therefore determined the DNA repair capacity through the level of persistent DNA damage, both via DNA breaks and oxidative DNA lesions, after 24 h posttreatment (Figures 3(a) and 3(b), representative images in Supplementary Material 1). The analysis of the repair rate is shown in Figures 3(c) and 3(d). Notably, in hADMSCs, the DNA breaks induced by H_2_O_2_ were repaired more efficiently than oxidative lesions (77% versus 45% repair for DNA breaks and oxidative DNA lesions, respectively, upon exposure to 200 *μ*M H_2_O_2_) (Figures 3(c) and 3(d)). Notably, 24 h after treatment with 100 *μ*M H_2_O_2_, a concentration that could be reached in engrafted MSCs, repair was complete for both types of lesions.

### 3.3. DNA Breaks and Oxidative Lesion Accumulation through Adipocyte Differentiation

**Figure 4:**
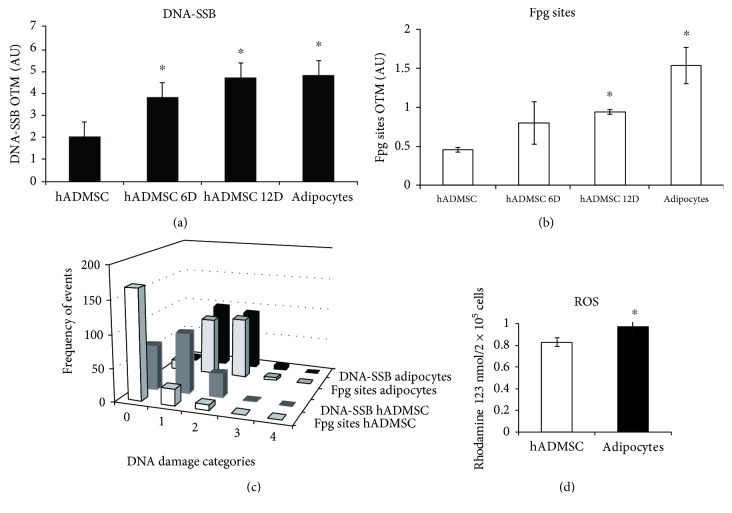
DNA damage accumulation through adipocyte differentiation of hADMSCs. DNA damage corresponding to DNA breaks and alkali labile sites accumulated over 14 days of adipocyte differentiation was expressed as Olive tail moment (OTM), (a) (mean ± SD). DNA oxidative damage accumulation, corresponding to DNA-Fpg sites (7,8-dihydro-8-oxoguanine (8-oxoguanine), 8-oxoadenine, aflatoxin B1-fapy-guanine, 5-hydroxy-cytosine, and 5-hydroxy-uracil) expressed as OTM, is represented in (b) (mean ± SD). Basal DNA damage (breaks + alkali labile sites) and basal DNA oxidative damage (Fpg-sensitive sites) of hADMSCs (U = undifferentiated) and adipocytes (D = differentiated) are represented in categories of damage (0, without damage to 4, highest damage) in (c). ROS intracellular levels in hADMSCs and adipocytes are shown in (d) (mean ± SD). Data represent 3 or 4 independent experiments with duplicates. Nonparametric tests were used for single cell gel electrophoresis analyses, Kruskal-Wallis (one-way ANOVA on ranks), and all pairwise multiple comparison procedures (Dunn's test). Differences versus hADMSCs (undifferentiated), ^∗^ *p* < 0.05. Images in Supplementary Figure 4.

DNA damage determined as breaks through adipocyte differentiation shows an increase as a function of the differentiation time with a significant increase already after 6 days (hADMSC 6D) (Figure 4(a), images in Supplementary Figure 2). Similarly, oxidative DNA lesions also increased, although this increase was significant in the final stages of differentiation, since day 12, and to the complete differentiation (14 days) (Figure 4(b), image in Supplementary Figure 2). As observed in the case of hADMSCs, the level of DNA breaks is relatively higher than that of Fpg-sensitive sites. Additionally, we demonstrate the contrast between the basal DNA damage and oxidative lesions according to categories showing undifferentiated (U) and terminally differentiated adipocytes (D) (Figure 4(c)) and increment of ROS levels (Figure 4(d), comet images in Supplementary Figure 2).

### 3.4. H_2_O_2_-Induced DNA Damage and Repair Capacity through Adipocyte Differentiation

**Figure 5:**
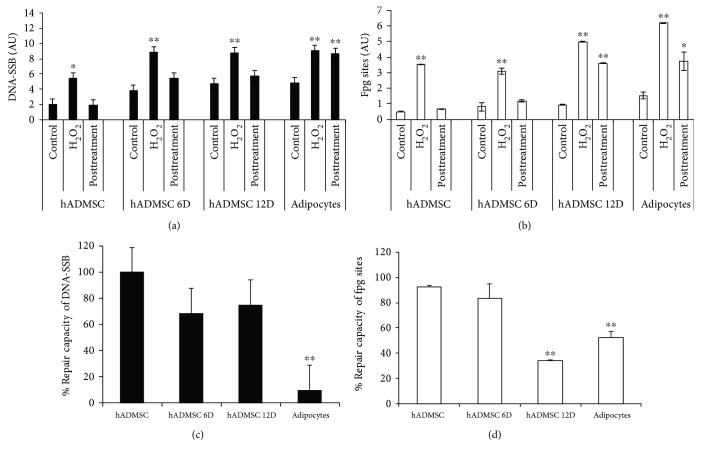
H_2_O_2_-related DNA damage induction and DNA kinetic repair through adipocyte differentiation of hADMSCs. DNA breaks and alkali labile sites are represented in (a) (mean ± SD); DNA-Fpg sites (7,8-dihydro-8-oxoguanine (8-oxoguanine), 8-oxoadenine, aflatoxin B1-fapy-guanine, 5-hydroxy-cytosine, and 5-hydroxy-uracil) are shown in (b) (mean ± SD). Data represent 3 or 4 independent experiments with duplicates. Nonparametric tests were used for single cell gel electrophoresis analyses, the Kruskal-Wallis test employed one-way ANOVA on ranks, and all pairwise multiple comparison procedures used Dunn's test. Differences versus controls correspond to the differentiation stage, ^∗^ *p* < 0.05 and ^∗∗^ *p* < 0.01. The repair capacity of DNA breaks and alkali labile sites is shown in (c) (mean ± SD). The repair capacity of DNA-Fpg sites (7,8-dihydro-8-oxoguanine (8-oxoguanine), 8-oxoadenine, aflatoxin B1-fapy-guanine, 5-hydroxy-cytosine, and 5-hydroxy-uracil) is shown in (d) (mean ± SD). Student's *t*-test versus hADMSCs, ^∗^ *p* < 0.05 and ^∗∗^ *p* < 0.01. Images in Supplementary Figure 5.

The relevance of the DNA damage accumulated through adipocyte differentiation was evaluated by the adipocyte response to H_2_O_2_ and the measurement of DNA repair capacity 24 h after treatment. The exposure to 100 *μ*M H_2_O_2_ does not affect hADMSC viability (Table 1); therefore, this experimental condition was selected to monitor DNA damage induction and repair (Figures 5(a) and 5(b), images in Supplementary Figure 3). The capacity of H_2_O_2_ to induce DNA breaks has shown similar behavior during differentiation even though the values were higher than in undifferentiated hADMSCs (Figure 5(a)); meanwhile, the capacity of H_2_O_2_ to induce DNA oxidative lesions was higher only in the latest stages of adipocyte differentiation (hADMSC 12D and adipocytes) (Figure 5(b)).

The DNA repair capacity indicates that while breaks are fully repaired in undifferentiated hADMSCs, repairs of this type of damage in adipocytes drop drastically to 10% (Figure 5(c)). Less dramatic changes were observed for the repair of oxidative lesions with repair rates of 92% and 50% in undifferentiated hADMSCs and adipocytes, respectively (Figure 5(d)).

## 4. Discussion

hADMSCs represent a potential source of MSCs for cell therapy [4]. As expected, these cells maintain the ability to be differentiated into chondrocytes, osteoblasts, or adipocytes, as shown by the expression of specific surface markers [11, 14]. Moreover, they express the human fatty acid transport protein-1 (hFATP-1), which is stimulated by insulin and facilitates the transport of fatty acids across the cell membrane to promote the accumulation of long chain fatty acids (LCFAs). This explains the formation of fat vesicles during adipocyte differentiation (Figure 1). Notably, we found an increase in the expression of CD146^−^ in hADMSCs compared with adipocytes, which is also known as melanoma adhesion molecule and could account for the increased adhesion capacity characteristics of the adipocytes. DNA damage is expected to be efficiently removed in stem cells [15, 16], and indeed, hADMSCs efficiently repair DNA breaks and oxidative DNA lesions. Our results were obtained using an early cell passage (4th passage) and are in line with other studies that employed adipose-derived stem cells obtained by bone marrow or femoral/abdominal liposuction [17, 18].

To provide new insights into the use of hADMSC in cell therapy, we investigated its response to oxidative damage generated in DNA induced by a physiological flow of hydrogen peroxide. Experimentally *in vitro*, the reproduction of the flow of hydrogen peroxide is complex; however, it is closer to what cells *in vivo* are likely to experience. In this work, we only studied the effect triggered by a single dose of 100 *μ*M. These cells, as well as their differentiated counterpart, appear to be relatively resistant to H_2_O_2_ because no cytotoxicity was observed up to 100 *μ*M (Table 1 and Figure 2(a)), a dose that is cytotoxic in a wide variety of animals, plants, and bacterial cells in culture [8, 13]. In terms of DNA repair capacity, hADMSCs are fully proficient in the repair of breaks and oxidative lesions induced by hydrogen peroxide levels up to 100 *μ*M. Above this dose (>100 *μ*M), a significant inhibition of DNA repair is observed (Figure 3), suggesting that the control of the ROS environment is a key factor for stem cell survival. Some studies report that stem cells and MSCs require DSB (double strand break) repair to address DNA damage [13, 15, 18]. Khan et al. recently suggested that only cells with an efficient response to DNA damage are allowed to enter adipogenic differentiation; they proposed that SNEV (senescence evasion factor) regulates adipogenesis, acting as a checkpoint for DNA damage accumulation in human adipocyte-derived stem cells after acute treatment with hydrogen peroxide [17]. A cross talk between DDR and differentiation would therefore provide stem cells with a stringent control of genetic stability before undergoing self-renewal and differentiation [18, 19]. Here, we addressed how hADMSCs respond to an oxidative insult (H_2_O_2_ 100 *μ*M) when proliferating and during adipocyte differentiation. The response to endogenous DNA damage as well as to H_2_O_2_-induced DNA damage reflects a gradual decrease in repair capacity during the differentiation program. This decrease in repair capacity is more marked than the changes in intracellular ROS levels, which are only slightly modified. Similarly, a decrease in the repair capacity of oxidative damage has been previously reported during myogenesis as well as during neuronal differentiation [13, 20]. Additionally, we want to emphasize that our protocol only reflects the sites sensitive to the Fpg, since we only do the enzymatic digestion immediately after the cell lysis and perform the electrophoretic shift; that is, we do not perform alkaline incubation for unwinding; this avoids the generation of AP sites together with the oxidative lesions digested by the Fpg. When the repair capacity along adipogenesis is analyzed as a function of the DNA lesion type, an interesting scenario emerges: while DNA break repair is fully impaired, the removal of Fpg-sensitive sites is decreased but remains active (50% reduction) in adipocytes. Diverse DNA base lesions are removed by specific repair enzymes to avoid harmful consequences. Abasic sites are repaired by APE1 to avoid mutagenic bypass, replication fork stalling, or conversion to double strand breaks. 7-Methylguanine and 3-methyladenine are repaired by N-methylpurine DNA glycosylase to generate abasic site formation, ring opening to FaPy-G, replication stalling, or chromosomal instability. SSB are repaired by the single strand break repair (SSBR) machinery to avoid replication fork collapse or conversion to DSB. 8-Oxoguanine is the most common and mutagenic oxidized DNA lesion and is repaired by OGG1 to avoid mutagenic base mispairing. However, oxidatively generated DNA lesions are not only a mutagenic risk factor but also a regulatory marker (for a review, see [21]). Therefore, the accumulation of DNA oxidative lesions may also be of great concern for cells that do not replicate, such as terminally differentiated adipocytes that have to maintain the integrity of the genes essential for their function. This may explain why the repair of oxidatively generated DNA lesions remains active (although reduced) in adipocytes. Whether the transcription-coupled repair of oxidized base lesions [22, 23] is involved warrants further investigation.

## 5. Conclusions

In regenerative medicine, the use of human adipose-derived mesenchymal stem cells is extensive; however, little is known regarding their response to DNA damage and in particular to the reactive oxygen species (ROS) that are present in the microenvironment of implantation. The role of these ROS in the microenvironment can be partly addressed experimentally by the treatment of a hydrogen peroxide bolus. The present study is the first evidence of DNA breaks and oxidative DNA damage accumulated through adipogenesis, in concordance with the increase of adipogenic biomarkers such as hFATP-1, CD 146, and lipid droplet formation. In addition, we show the increased vulnerability to hydrogen peroxide in the adipogenic differentiation and some interesting particularities in DNA repair capacity which emerge depending on the specific DNA lesions. While DNA break repair is fully impaired, the removal of Fpg-sensitive sites is decreased but remains active (50% reduction) in adipocytes. The removal of oxidative DNA lesions has a primarily protective function; however, our proposal is that all remaining Fpg-sensitive sites realize the regulatory functions in signal transduction and transcriptional regulation required by specialized cells, such as terminally differentiated adipocytes.

## Figures and Tables

**Table 1:** Cell viability after H_2_O_2_ treatment (2 h) in hADMSCs through adipocyte differentiation and 24 h posttreatment.

	Control	H_2_O_2_ (100 *μ*M)	Posttreatment
hADMSC	96 ± 1	89 ± 4	92 ± 3
hADMSC 6D	98 ± 0	88 ± 1	89 ± 1
hADMSC 12D	99 ± 0.5	85 ± 0.5^∗^	85 ± 0^∗^
Adipocytes	95 ± 1	80 ± 1^∗^	86 ± 0.5^∗^

Mean ± SD, Student's *t*-test versus control ^∗^ *p* < 0.05.

## Supplementary Materials

Supplementary Materials Supplementary Figure 1: H_2_O_2_-induced DNA damage and repair capacity in hADMSCs. 2 h hydrogen peroxide treatment using 0 to 200 *μ*M and repair capacity presented as DNA damage remnant after 24 h posttreatment. Comet images at 4x magnification acquired by Komet 5.0 (Kinetic Imaging Ltd.). Methodological details are shown in the Material and Methods section. Supplementary Figure 2: DNA damage accumulation through adipocyte differentiation of hADMSCs. Differentiation follow-up at day 0 = hADMSC, day 6 = hADMSC 6D, day 12 = hADMSC 12D, and day 14 = adipocytes. Comet images at 4x magnification acquired by Komet 5.0 (Kinetic Imaging Ltd.). Methodological details are shown in the Material and Methods section. Supplementary Figure 3: H_2_O_2_-induced DNA damage and repair capacity through adipocyte differentiation. Hydrogen peroxide treatment using 100 *μ*M during 2 h and repair capacity presented as DNA damage remnant after 24 h posttreatment. Differentiation follow-up at day 0 = hADMSC, day 6 = hADMSC 6D, day 12 = hADMSC 12D, and day 14 = adipocytes. Comet images at 4x magnification acquired by Komet 5.0 (Kinetic Imaging Ltd.). Methodological details are shown in the Material and Methods section.
