# Supplementary material for: Hydrogen Peroxide-Induced DNA Damage and Repair through the Differentiation of Human Adipose-Derived Mesenchymal Stem Cells
Source: Stem Cells Int. 2018 Oct 10;2018:1615497. doi: 10.1155/2018/1615497 (PMC6199883; doi:10.1155/2018/1615497)

Supplementary Material

Supplementary figure 1. H_2O_2-induced DNA damage and repair capacity in hADMSCs. 2 h hydrogen peroxide treatment 0 to 200 µM, and repair capacity presented as DNA damage remnant after 24 h post-treatment. Comet images 4x acquired by Komet 5.0 (Kinetic images). Methodological details in material and methods section.

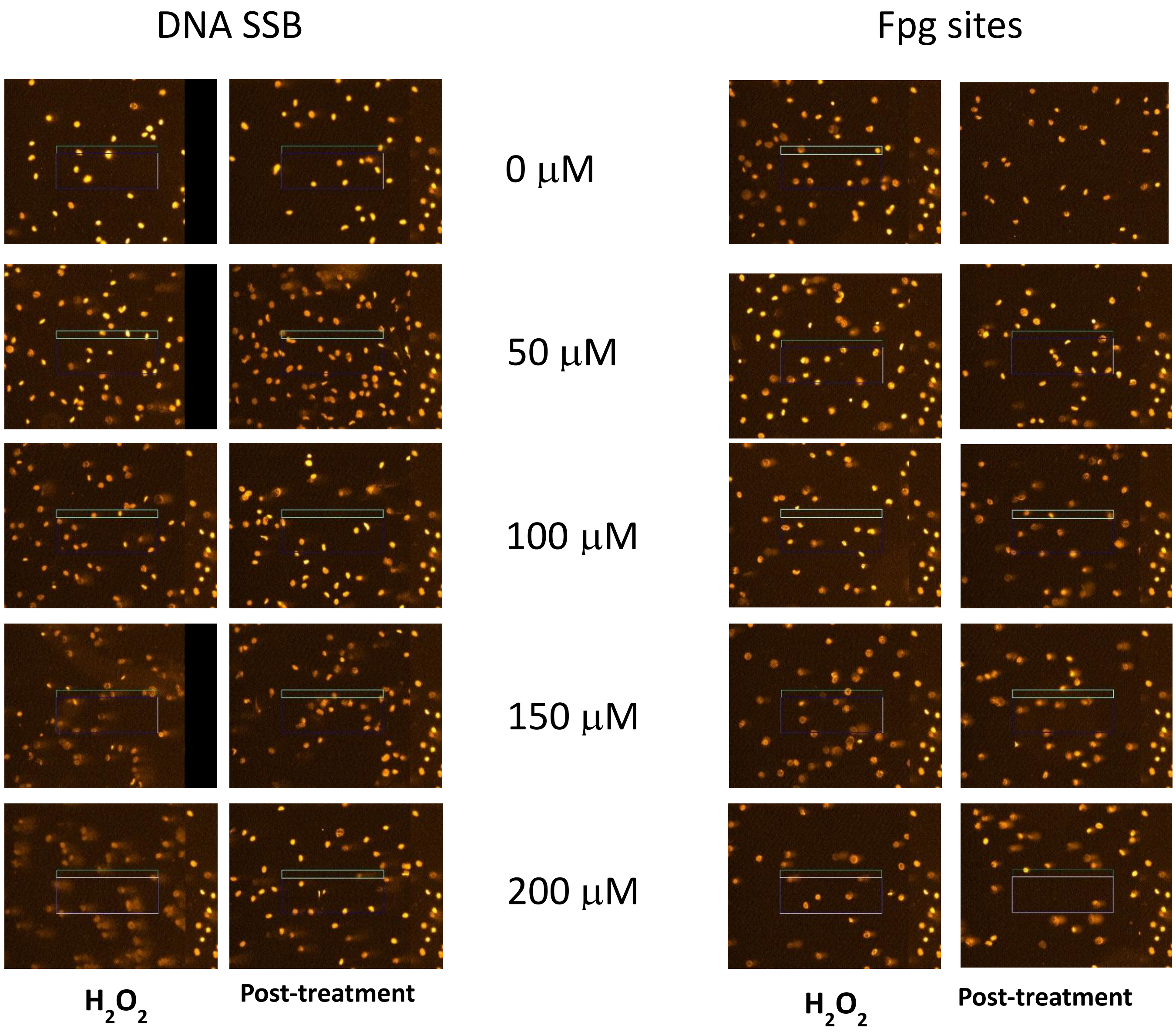

Supplementary figure 2. DNA damage accumulation trough adipocyte differentiation of hADMSCs. Differentiation follow-up at day 0= hADMSC, day 6= hADMSC 6D, day 12= hADMSC 12D and day14= Adipocytes. Comet images 4x acquired by Komet 5.0 (Kinetic images). Methodological details in material and methods section.

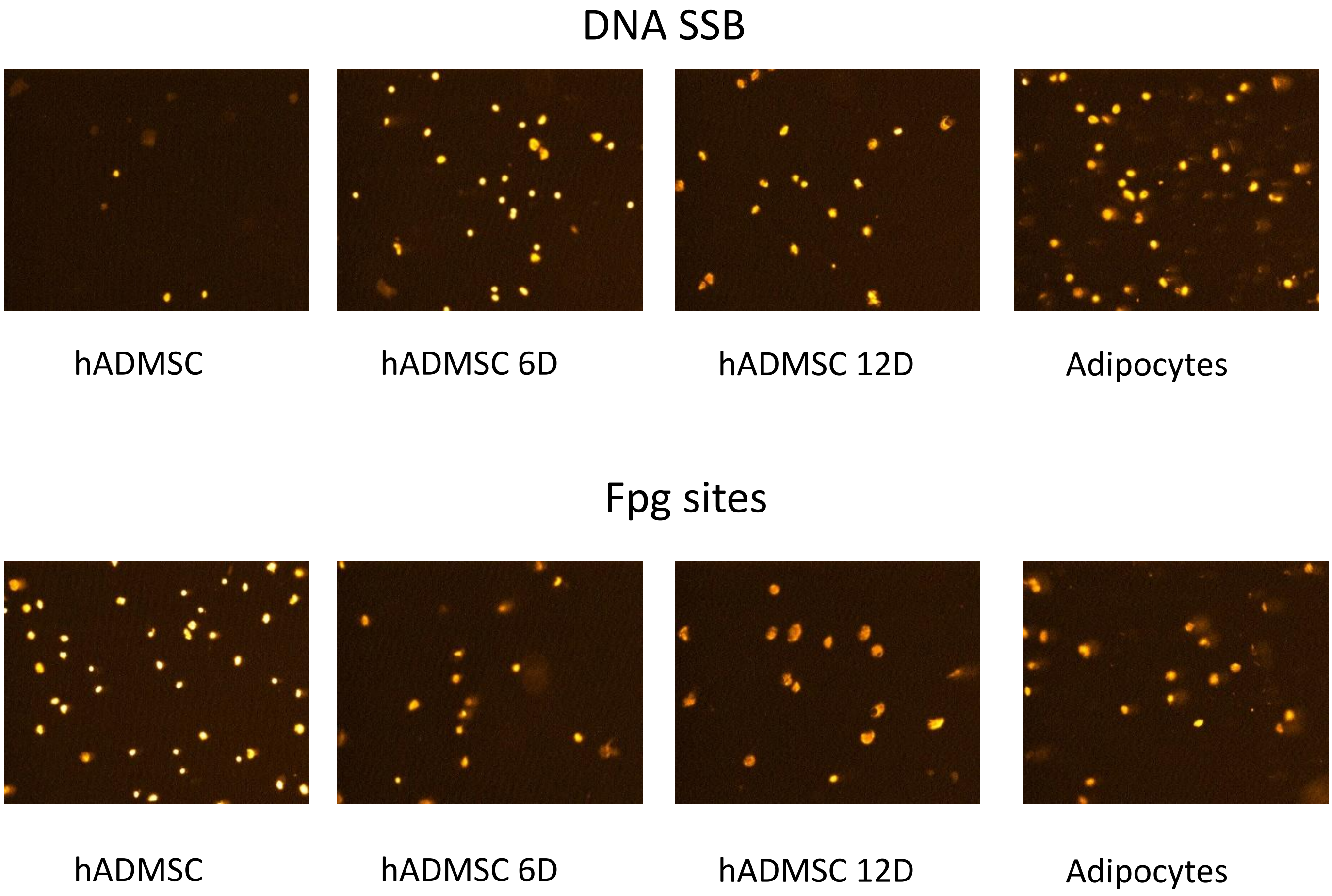

Supplementary figure 3. H_2O_2-induced DNA damage and repair capacity through adipocyte differentiation. Hydrogen peroxide 100 µM during 2h, and repair capacity presented as DNA damage remnant after 24 h post-treatment. Differentiation follow-up at day 0= hADMSC, day 6= hADMSC 6D, day 12= hADMSC 12D and day14= Adipocytes. Comet images 4x acquired by Komet 5.0 (Kinetic images). Methodological details in material and methods section.

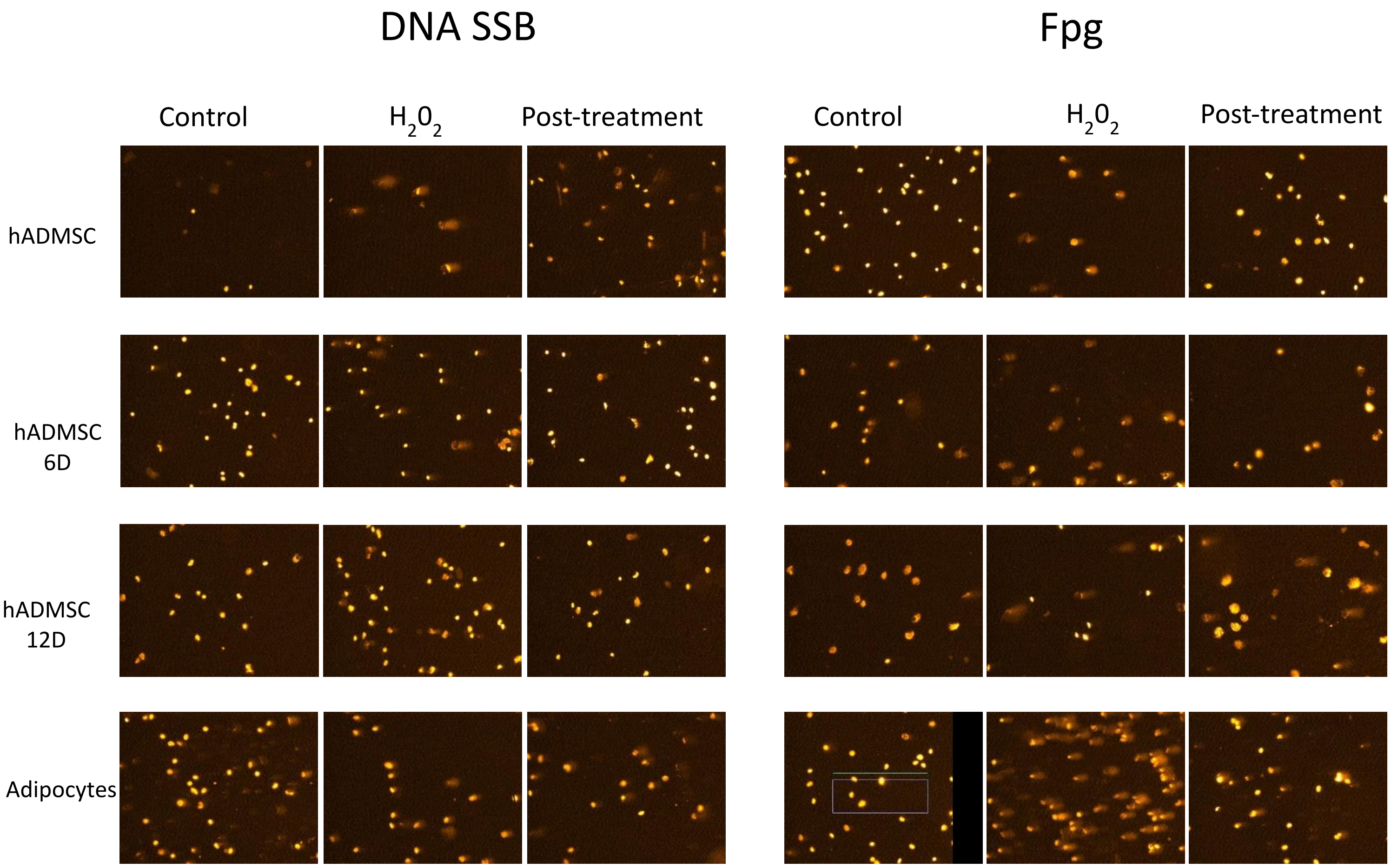

Supplement: Supplementary Materials — Supplementary Figure 1: H2O2-induced DNA damage and repair capacity in hADMSCs. 2 h hydrogen peroxide treatment using 0 to 200 μM and repair capacity presented as DNA damage remnant after 24 h posttreatment. Comet images at 4x magnification acquired by Komet 5.0 (Kinetic Imaging Ltd.). Methodological details are shown in the Material and Methods section. Supplementary Figure 2: DNA damage accumulation through adipocyte differentiation of hADMSCs. Differentiation follow-up at day 0 = hADMSC, day 6 = hADMSC 6D, day 12 = hADMSC 12D, and day 14 = adipocytes. Comet images at 4x magnification acquired by Komet 5.0 (Kinetic Imaging Ltd.). Methodological details are shown in the Material and Methods section. Supplementary Figure 3: H2O2-induced DNA damage and repair capacity through adipocyte differentiation. Hydrogen peroxide treatment using 100 μM during 2 h and repair capacity presented as DNA damage remnant after 24 h posttreatment. Differentiation follow-up at day 0 = hADMSC, day 6 = hADMSC 6D, day 12 = hADMSC 12D, and day 14 = adipocytes. Comet images at 4x magnification acquired by Komet 5.0 (Kinetic Imaging Ltd.). Methodological details are shown in the Material and Methods section. [file 1615497.f1.pdf]
